# Outcomes of a Medically Supervised Fasting Module on Healthy Females in a Controlled Residential Environment: A Brief Report

**DOI:** 10.7812/TPP/21.086

**Published:** 2021-12-06

**Authors:** Dhananjay Arankalle, Gulab Rai Tewani, Pradeep MK Nair, Jon Wardle

**Affiliations:** ^1^ Indian Naturopathy and Yoga Graduates’ Medical Association, Maharashtra, India; ^2^ Sant Hirdaram Yoga and Nature Cure Hospital and Medical College, Bhopal, India; ^3^ National Center for Naturopathic Medicine, Sydney, Australia

**Keywords:** vitamin D, fasting, metabolism, vitamin B12, nutrition, calorie restriction

## Abstract

**Background:**

Fasting is being used as a therapeutic and cultural practice for millennia. There are numerous reports available on beneficial effects of fasting on various disease conditions. Despite the mounting evidence on fasting, little is known on its physiological effects in humans as most of the studies on physiological effects are done in animals.

**Methods:**

Twenty healthy female volunteers (mean age ± SD, 21.95 ± 2.52 years) participated in a 10-day fasting program, which has 1 preparatory day and 1 refeeding day (1,000 kcal) and 8 fasting days (500 kcal). All the participants consented to participate in the study. Blood parameters like complete blood count, renal function test, total iron binding capacity, lipid profile, liver profile, vitamins D and B12, thyroid function tests, glycated hemoglobin, and air blood gas test along with anthropometric measurements were taken on the first and last day.

**Results:**

All the parameters under the study have shown statistically significant changes (p < 0.05) except hemoglobin (p = 0.7) and non-high density lipoprotein (p = 0.32). Notable changes were the significant increase in vitamins D and B12 levels that signifies the homoeostatic potential of a fasting regimen.

**Conclusion:**

The result depicts the positive impact of fasting on various physiological parameters that warrants further studies on the safety of fasting in diverse diseases, especially the ones that have metabolic disarray as the root cause. Despite the limitation of the smaller sample size and lack of a control group, the results are encouraging to devise disease-specific fasting programs.

## INTRODUCTION

Fasting and its effect on human metabolism are gaining importance among the physicians and other scientific fraternities. Over millennia, it has evolved as a potential tool to mitigate various diseases, which was once considered just as a superstitious religious practice. Historically, therapeutic fasting was well represented in traditional medical practice, but its use had fallen out of favor with the move to more pharmacologically focused therapies in the past 50 years. However, anecdotal reports suggest that in recent times, the promotion of fasting as a therapeutic intervention has increased, and the few studies that have examined such use (from Germany and the United States, albeit each from over 10 years ago) indicate that it is gaining in popularity as a self-care method for prevention and health promotion.^1^^,^^2^

In therapeutic fasting, a nutritional intake below 500 kcal/day is aimed for, as this has been shown to induce strong neuroendocrine and metabolic responses, which are hypothesized to improve health.^2^^,^^3^ Caloric restriction and alternate-day fasting appear to be associated with prevention of some chronic diseases.^2^^,^^4^ Therapeutic fasting therapy has been shown to alleviate symptom burden in rheumatoid arthritis,^5^ reduce the risk of coronary artery disease and diabetes mellitus,^6^^–^^8^ and has been argued to generally improve mood disorders.^9^^,^^10^ In experimental research, there is also promising evidence that fasting and/or caloric restriction delay aging and the onset of age-related and neurodegenerative diseases.^11^^,^^12^

Epidemiological data also suggest that fasting is being used for a wide variety of health conditions, not necessarily those with the strongest evidence base.^13^ However, despite the popularity and potential promise of therapeutic fasting as a preventive measure, little detail is known about the specific physiological responses to fasting in controlled and deliberate settings, either in terms of those that are clinically beneficial or those that may present safety issues. This information is essential to identifying potential opportunities and problems for therapeutic fasting. In direct response to this research gap, the study presented here aims to identify the physiological impact on a large variety of parameters of a 1-week controlled-fasting regime.

## MATERIALS AND METHODS

The study was conducted at a yoga and naturopathy medical college in India. The participants were 20 healthy female volunteers (N = 20) who volunteered to participate in this study. The study protocol was approved by the institutional ethics committee of the medical college. The fasting regimen was conducted as a part of the academic program for the undergraduate medical students in yoga and naturopathy. A written informed consent was obtained from all the participants enrolled in this study. Physical examinations were performed on all the participants, including the measurement of blood pressure (at least twice), body weight, and height. All the participants were given individual diaries to record the daily events in response to fasting.

### Fasting Protocol

The fasting regimen consisted of a total of 10 days, which included 2 days of normal diet (approximately 1,000 kcal) at the beginning and end of the fasting for preparatory and re-introductory purpose. A cold water enema was given to all the participants to clean the intestinal tract before starting the fast. The total fasting period was for 8 days, where all the participants had taken thick vegetable juices or lemon honey juice. The total calorie intake was restricted to 500 kcal. The details of the diet provided are shown in Table 1. Along with the diet, all the participants were provided with supportive eliminative therapies like hip bath, cold water enema, and mud packs to the abdomen and eyes. These therapies were intended to provide comfort to the participants. All the participants stayed on the naturopathy hospital campus and were under the supervision of licensed yoga and naturopathy physicians.

**Table 1.** Fasting diet regimen

Day of fasting	Diet provided	Approximate calories (per day)
Day 1 and day 10	Holy basil herbal tea (100 mL), lemon honey water (600 mL) Cucumber juice (200 mL) Boiled green gram sprouts (75 g) Rice and lentil porridge (100 g) Plain vegetable soup (150 mL) Water (3 L)	1,000 kcal
Day 2 to day 9	Holy basil herbal tea (100 mL) Lemon honey water (1,000 mL) Water (3 L)	500 kcal

### Outcome Measures

Blood samples were collected twice from all the participants, one sample at the beginning of day 1 and another at the end of day 10. Complete blood count, renal function test, total iron binding capacity, lipid profile, liver profile, vitamins D and B12, thyroid function tests, glycated hemoglobin (HbA_1c_), and air blood gas test were done once before and once after the fasting period.

### Statistical Analysis

All the data were analyzed using Statistical Package for the Social Sciences (SPSS) version 20.0. The statistical analysis was primarily descriptive, and the statistical significance of changes between baseline and follow-up was determined on an exploratory basis. A repeated measure analysis of variance was used to assess any changes with participants over time for multiple observations.

## Results

All 20 of the participants completed the study. The participants were aged between 20 and 32 years (mean age ± SD, 21.95 ± 2.52 years). All the parameters under the study showed statistically significant changes except hemoglobin and non-high density lipoprotein. A statistically significant reduction in platelet count was also observed in the participants. A significant reduction in weight (p = 0.0005) and body mass index (BMI) (p = 0.0004) was observed. An average weight reduction of 3.5 kg from the baseline was noted in this fasting program. A similar reduction was seen in blood pressure as well. The mean values of the blood pressure decreased significantly from 118 ± 2 mmHg to 110.6 ± 0.4 mmHg for systolic blood pressure (p < 0.001) and from 78.4 ± 2.2 mmHg to 74 ± 1 mmHg for diastolic blood pressure (p < 0.001). The results are shown in Table 2. No adverse events were reported by the participants except for mild headache and lethargy, which are expected reactions/responses during the first day of fasting.

**Table 2.** Summary of changes in physiological and metabolic blood parameters pre- and post-fasting regimen

Test (units)	Baseline (± SD)	Post-intervention (± SD)	Difference (95% CI)	p value
CBC and blood sugar				
FBS^a^ (mg/dL)	85.20 (± 8.33)	75.65 (± 3.65)	−9.55 (−5.01 to −14.09)	< 0.001
TLC^a^ (× 103/µL)	7.93 (± 2.07)	6.23 (± 2.22)	−1.70 (−0.91 to −2.48)	< 0.001
Hemoglobin (g/dL)	12.17 (± 1.08)	12.13 (± 0.98)	−0.04 (−0.22 to 0.29)	0.780
MCV^a^ (fL/cell)	90.81 (± 8.85)	93.02 (± 9.37)	2.21 (1.33-3.08)	< 0.001
MCH (pg)	28.30 (± 3.79)	28.16 (± 3.63)	0.14 (−0.01 to 0.28)	0.070
RDW-SD^a^ (%)	49.13 (± 4.61)	52.43 (± 4.24)	3.30 (2.44-4.17)	< 0.001
Platelets^a^ (× 103/µL)	292.05 (± 67.95)	246.07 (± 64.69)	−45.35 (−22.30 to −68.40)	0.001
PCT^a^ (%)	0.31 (± 0.06)	0.28 (± 0.06)	−0.03 (−0.00 to −0.01)	0.025
Iron (µg/dL)				
TIBC^a^ (µg/dL)	394.96 (± 44.53)	366.95 (± 49.30)	−28.01 (−15.32 to 40.70)	< 0.001
Renal profile				
Calcium^a^ (mg/dL)	8.95 (± 0.24)	9.33 (± 0.26)	0.38 (0.27-0.49)	< 0.001
Uric acid^a^ (mg/dL)	4.33 (± 0.78)	5.90 (± 1.56)	1.57 (1.01-2.12)	< 0.001
Lipid profile				
TC^a^ (mg/dL)	160.40 (± 33.86)	149.20 (± 32.66)	−11.20 (−2.24 to −20.16)	0.017
HDL^a^ (mg/dL)	48.35 (± 9.40)	40.75 (± 7.36)	−7.60 (−4.34 to −10.85)	< 0.001
LDL^a^ (mg/dL)	96.20 (± 28.90)	80.60 (± 26.71)	−15.60 (−8.63 to −22.57)	< 0.001
TC ratio^a^	3.44 (± 0.96)	3.75 (± 0.95)	−0.32 (−0.14 to −0.49)	0.001
Non-HDL (mg/dL)	112.06 (± 34.86)	108.63 (± 33.16)	3.43 (−10.54 to 3.69)	0.326
Liver profile				
ALKP^a^ (IU/L)	79.11 (± 17.61)	71.29 (± 15.45)	−7.82 (−4.82 to −10.80)	< 0.001
Bilirubin^a^ (mg/dL)	0.58 (± 0.19)	0.68 (± 0.22)	0.08 (0.00-0.15)	0.042
Protein^a^ (g/dL)	7.41 (± 0.26)	7.64 (± 0.38)	0.23 (0.04-0.42)	0.021
Serum albumin^a^ (g/dL)	4.23 (± 0.18)	4.37 (± 0.19)	0.15 (0.05-0.25)	0.006
Vitamins				
Vitamin D^a^ (ng/mL)	7.75 (± 2.00)	9.47 (± 2.23)	1.72 (1.00-4.98)	< 0.001
Vitamin B12^a^ (pg/mL)	199.44 (± 49.67)	253.89 (± 91.09)	54.44 (28.39-80.50)	< 0.001
Thyroid				
T3^a^ (ng/dL)	112.00 (± 15.52)	97.60 (± 17.44)	−14.40 (−9.26 to −19.54)	< 0.001
T4^a^ (µg/dL)	7.68 (± 1.11)	8.53 (± 1.04)	0.85 (0.46 to 1.24)	< 0.001
Diabetes				
HbA_1c_^a^ (%)	5.31 (± 0.41)	5.06 (± 0.43)	−0.25 (−0.20 to −0.30)	< 0.001
ABG^a^ (mg/dL)	105.50 (± 11.81)	98.35 (± 12.44)	−7.15 (−5.60 to −8.70)	< 0.001

a Significant p value < 0.05.

ABG = arterial blood gas; ALKP = alkaline phosphatase; CBC = complete blood count; FBS = fasting blood sugar; HbA_1c_ = glycated hemoglobin; HDL = high density lipoproteins; TLC = total leukocyte count; MCV = mean corpuscular volume; MCH = mean cell hemoglobin; RDW-SD = red blood cell distribution width-SD; PCT = plateletcrit; TIBC = total iron binding capacity; LDL = low density lipoproteins; TC = total cholesterol; T3 = triiodothyronine; T4 = thyroxine;.

## DISCUSSION

As reported above, fasting for 8 days was safe and well tolerated by the participants. Besides this, it improved the health parameters under study significantly, indicating the favorable physiological changes post fasting. Similar to the previous studies,^14^ our participants also showed marked weight loss and reduced BMI, which gives a clear indication that periodic fasting may help in reducing the risk factors for metabolic disorders. However, the authors do not intend to make any recommendations with the current data, as long-term follow-ups are warranted for establishing the impact of fasting on weight and BMI. The cardioprotective effect of fasting was demonstrated by the significant changes observed in lipid profile. The changes observed in the renal profile, thyroid function, and liver function can be attributed to enhanced elimination and the homoeostatic effects of fasting. Another remarkable change observed was the increase vitamin D and B12 levels. There exists a relationship between body weight and vitamin D levels.^15^ A hypocaloric interventional study reported that a 10% weight reduction can increase the level of vitamin D (25-hydroxyvitamin D) by 34% without any additional supplementation of vitamin D.^16^

Further, the reduction in HbA_1c_ and fasting blood glucose levels strengthen the fact of an interlink between glucoregulation,^17^ vitamin D status,^16^ and weight reduction.^14^ A lowered vitamin D level is further associated with increased adiposity, which triggers insulin resistance.^18^ Hence, it is a plausible fact that fasting can be a holistic solution for mitigating noncommunicable disease owing to its positive impact on glucoregulation, increased vitamin D levels and, reduction in adiposity. The rise in vitamin B12 levels should be viewed as a sign of the pluralistic protective effect of fasting on cardio-metabolic risk factors, as lowered serum vitamin B12 is associated with increased BMI and adverse cardio-metabolic outcomes.^19^ The present study has reported marked reduction in weight and BMI, which could be the plausible reason for the clinically meaningful changes in these vitamin levels.

**Figure 1.**
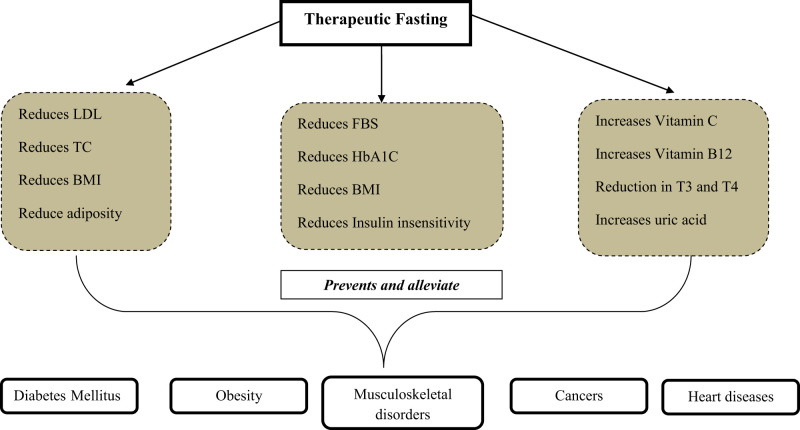
Plausible outcomes and metabolic changes of fasting. FBS = fasting blood sugar; LDL = low density lipoproteins; TC = total cholesterol; T3 = triiodothyronine; T4 = thyroxine; HbA_1c_ = glycated hemoglobin; BMI = body mass index.

Our study also exhibited a significant reduction in platelet count, similar to the results of previous studies.^20^^,^^21^ This reduction may be due to a deficient supply of micronutrients like iron or other vitamins during fasting.^22^ A pictorial representation of the physiological and metabolic changes of fasting based on our results is depicted in Figure 1. Nevertheless, the present study has demonstrable limitations. The results presented here are on healthy volunteers, which may not be generalizable among diseased population due to their medications or the chronicity of the disease. The utility of fasting among individuals with chronic pathologies needs to be examined through future studies. Further, the translational value of the present inferences needs to be cautiously examined, as the current data report the changes in clinical parameters for an 8-day fasting period followed by a single day of refeeding. Whether these changes are sustainable can only be established if these changes persist once the refeeding is established for a longer time. This limits the authors from making any clinical recommendations at this point in time.

Further, fasting therapy as practiced by Indian yoga and naturopathy physicians is more of a detoxification therapy, which includes minor cleansing therapies like hip bath, cold water enema, and mud packs to the abdomen and eyes. This is intended to stimulate the excretory systems, as encouraged by the international fasting consensus.^23^ However, these therapies may have an influence on the outcomes, which is not contemplated in the present study. This may be a potential confounder to the results, which needs to be considered in future studies.

A relatively large sample size and the presence of a control group would have improved the generalizability of our results, which remains as a limitation of this study. However, the results are promising and favor the implication of fasting as a safe and effective method in different clinical scenarios.

## CONCLUSION

Our data suggest that therapeutic fasting for 8 days is safe and beneficial with numerous therapeutic benefits to offer. The discernible physiological and metabolic effects induced by fasting should be carefully evaluated by future studies to provide clinical insights in treating specific diseases.
